# Role of NLRP3 Inflammasomes in Disorders of Children’s Digestive Systems: A Narrative Review

**DOI:** 10.3390/pediatric17050103

**Published:** 2025-10-07

**Authors:** Safaa ELMeneza

**Affiliations:** Faculty of Medicine for Girls, Al-Azhar University, Cairo 4434103, Egypt; safaaelmeneza@azhar.edu.eg

**Keywords:** inflammasomes NLRP3, neonates, pediatrics, gastrointestinal and hepatic diseases, NLRP3 inhibitors, gap NLRP3

## Abstract

Background/Objectives: This review article highlights the role of the nucleotide-binding domain, leucine-rich repeat, pyrin domain-containing 3 protein (NLRP3) inflammasomes in various gastrointestinal and hepatic disorders in the pediatric age group. NLRP3 inflammasomes are one of the principal intracellular innate immune sensors. During inflammation, molecules such as caspase-1 and the release of IL-1β and IL-18 are produced. The NLRP3 inflammasome participates in the preservation of intestinal homeostasis and mucosal immune response. The objective is to evaluate the published articles related to the role of NLRP3 inflammasomes in common pediatric gastrointestinal and hepatic disorders in order to identify the future perspective regarding their possible therapeutic values. Methods: We searched Medline for NLRP3 inflammasomes and disorders of the digestive system during childhood. Results: Although the majority of articles were related to various disorders of adults, such as Alzheimer’s disease, Parkinson’s disease, atherosclerosis, as well as neurodevelopmental disorders, such as schizophrenia, a few published datasets were related to the roles of NLRP3 in the pediatric age group: they addressed autism, rheumatoid arthritis, and other autoimmune diseases, as well as inflammatory bowel diseases (IBD) and hepatic infection. Some research demonstrated that the NLRP3 inflammasome has a protective role; however, it also has a pathogenic function. Conclusions: This review focused on the comprehensive role of inflammasome NLRP3 in the most common pediatric and neonatal gastrointestinal and hepatic diseases, including clinical and experimental studies, as well as the pharmacological inhibitors for NLRP3 inflammasomes, which may provide future therapy for GIT problems, such as IBD.

## 1. Introduction

Inflammasomes

In 2002, Jurg Tschopp was the first to project the term inflammasome when he described a high molecular weight nucleotide-binding domain complex, leucine-rich repeat-containing protein (NLR) family pyrin domain. The inflammasome is a multimeric protein complex; it is composed of three key groups: pattern-recognition receptors (PRR), NOD-like receptors (NLR), or AIM2-like receptors; poptosis-associated speck-like proteins containing caspase recruitment domains (ASCs); and 3 caspase proteases [1]. Its core role is to transfer the pro-IL-1b and pro-IL-18 to the final acting structures to implement inflammatory cell death, referred to as pyroptosis [2].

The innate immune system acts as a shield to protect the gastrointestinal tract from pathogenic microorganisms. The inflammasomes play a role in innate immunity, recognizing bacteria, fungi, viruses, and products of cell damage that trigger inflammatory responses through the release of pro-inflammatory factors [3]. Inflammasomes activate inflammatory mediators and induce cell death to protect against infectious agents while maintaining homeostasis.

Inflammasomes can identify pathogenic organisms and their metabolites that cause infection and inflammation, contributing to innate immunity [3]. Thus, inflammasomes protect the host from infectious agents and support homeostasis.

Both pathogen-associated molecular patterns (PAMPs) and damage-associated molecular patterns (DAMPs) provoke the inflammasome production that regulates the secretion of caspase-1; consequently, it controls the secretion of interleukin-1 (IL-1) and IL-18. Moreover, it can lead to inflammatory cell death, such as pyroptosis [2]. This indicates that inflammasomes play a dynamic function in innate and adaptive immunity, as well as inflammatory reactions in the host [4]. Previously, a number of inflammasomes have been recognized and broadly classified: NLRP1 inflammasome, NLRP3 inflammasome, NLRC4 inflammasome, NLRP6 inflammasome, and AIM2 inflammasome [2]. This review aims to evaluate the published articles related to the role of NLRP3 inflammasomes in common pediatric gastrointestinal (GIT) and hepatic disorders in order to identify future perspectives regarding their possible therapeutic values. This review also aims to contribute to the existing body of knowledge by presenting new findings, insights, methodologies, and perspectives on the role of NLRP3 inflammasomes in pediatric digestive system diseases. The expected impact is to contribute to knowledge and practical applications. We provide all the existing information related to the NLRP3 inflammasome in one review. It aims to assist in our understanding of the pathogenesis of GIT disorders. The review summarized the gaps in knowledge and topics that need future research. We decided to focus on the NLRP3 inflammasome because of its role as a key player in innate immunity and chronic inflammation. It is implicated in numerous diseases. Understanding its activation by various cellular and molecular events, as well as aberrant signaling, offers potential therapeutic targets for these conditions. The newly developed inhibitors can block the detrimental effects of NLRP3.

## 2. Materials and Methods

We conducted a search of the peer-reviewed, full-text published studies from electronic databases, including PMC and PubMed, applying various combinations of keywords, including NLRP3 inflammasomes and NLRP3 inflammasome role in intestinal mucosal immunity. We also used the following keywords: pathogenic roles of the NLRP3 inflammasome, protective role of the NLRP3 inflammasome, NLRP3 inflammasome in gastrointestinal diseases, and hepatic disorders in the pediatric age group. Experimental and clinical studies were also included. The correlated articles that were published between 2000 and January 2025 were incorporated. This narrative review provides a comprehensive background on the available data and information regarding the role of NLRP3 inflammasomes in pediatric digestive system disorders.

## 3. Discussion

### 3.1. Structure of NLRP3 Inflammasomes

The NLRP3 inflammasome has three original elements: NLRP3, ASC, and procaspase-1. These elements are composed of three proteins: a pyrin domain, a nucleotide-binding and oligomerization domain (NOD), and a leucine-rich repeat domain [5]. The activation of the NLRP3 inflammasome could occur through a two-step process: a reaction to several different stimuli resulting from microorganisms, various endogenous molecules, and cytokines that provoke macrophages to augment NLRP3 and pro-IL-1β extractions by generating the transcription factor NF-κB. The initial step involves priming by certain PAMPs or DAMPs, which stimulate the production of NLRP3 and the pro-form of IL-1β in affected cells. Other molecules that regulate NLRP3 and pro-IL-1β expressions include myeloid differentiation primary response protein 88 and TIR domain-containing adaptor-inducing interferon-β [6].

The subsequent step in NLRP3 inflammasome activation involves ligand-induced oligomerization of NLRP3, which facilitates the assembly of NLRP3, ASC, and procaspase-1. It was further described that inflammasome assembly is initiated by pyrin domain–pyrin domain interfaces involving NLRP3 and ASC, and the ATPase action of the NOD domain helps with NLRP3 oligomerization. He et al. described the canonical and noncanonical inflammasome pathways [7]. In the initial prototype, the NLRP3 inflammasome is primed by bacterial molecules or cytokines and is subsequently stimulated by a signal, such as ATP. This was suggested based on the detection of caspase-11-dependent pyroptosis in murine macrophages. In humans, the equivalents of caspase-11 are caspase-4 and caspase-5 [8].

The regulation of NLRP3 expression of NLRP3 expression may occur through priming signals that operate via transcription-independent pathways [5]. Additionally, lipopolysaccharides (LPS) can initiate acute priming without inducing NLRP3 expression. Moreover, the researchers showed that frequent signaling events at the molecular and cellular levels are activated by NLRP3 inflammasome agonists, such as ionic flux, reactive oxygen species production, and lysosomal damage [9].

**Figure 1:**
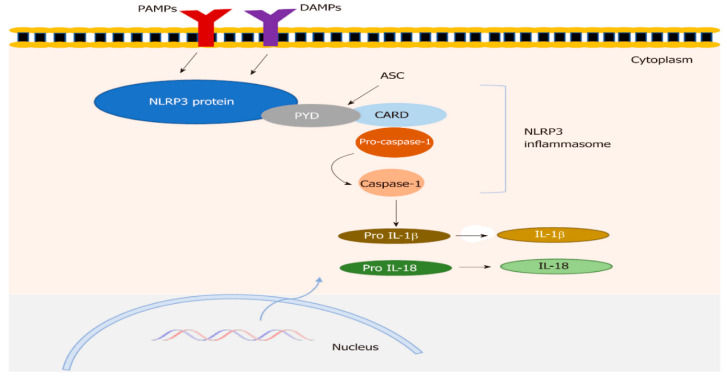
NLRP3 inflammasome structure; Tourkochristou E et al. [14].

Uninhibited activation of NLRP3 may be connected to diverse inflammatory diseases in humans, cryopyrin-associated periodic fever syndromes, rare hereditary auto-inflammatory diseases involving familial cold urticaria, Muckle-Wells syndrome, and neonatal-onset multisystem inflammatory disease and sepsis [10,11,12], intestinal inflammation, and ulcerative colitis [13]. Figure 1 shows the NLRP3 inflammasome structure.

### 3.2. NLRP3 Inflammasome in Neonates

In neonates, the function of the NLRP3 inflammasome is developmentally regulated, differing significantly from that of adults. A few studies have examined the NLRP3 inflammasome during intrauterine life and in neonates. Sharma et al. analyzed the extent of variability within the Toll-like receptor (TLR)/NLRP3 inflammasome pathways during gestational age. They reported the prevalence of immature low CD14-expressing/CD16pos monocytes in fetuses or neonates before 33 weeks of gestation, while high levels of pro-IL-1β are produced within high-CD14-expressing monocytes, even though these monocytes cannot express mature IL-1β. Although there is a lack of caspase-1 activity before 29 weeks of gestation, the production of the apoptosis-associated speck-like protein containing a CARD and the function of the P2 × 7 receptor are maintained. The study also suggests a role for fetal infection in regulating the activity of the NLRP3 inflammasome in utero and the important developmental mechanisms regulating IL-1β responses early in gestation, in part due to a downregulation of TLR-mediated NLRP3 expression. Such mechanisms may serve to limit potentially damaging inflammatory responses in a developing fetus [15,16].

A recent study by Wackerbarth et al. showed a decrease in the production and activity of NLRP3 inflammasome assembly in neonatal neutrophils in comparison to adult neutrophils, contributing to the reduced release of IL-1β upon stimulation with LPS/nigericin and reduced S100A8/A9 secretion following E-selectin activation [17]. Preterm infants showed reduced activation in NLRP3 inflammasome activity, too. However, rapid maturation after birth reaches adult levels during the early neonatal period.

The role of the impaired NLRP3 pathways in intra-amniotic inflammation and the preterm birth cascade, and fetal injury or early neonatal death was evidenced. The study of Motomura et al. precisely proves that the NLRP3 sensor molecule and inflammasome are important for activating intra-amniotic and decidual inflammation, fetal membrane activation, uterine contractility, and cervical dilation. NLRP3 signaling in the fetus and the mother is involved in the early initiation of the labor cascade. Pursuing the NLRP3 pathway might avert adverse perinatal outcomes [16].

The dysregulated NLRP3 activation is a critical mechanism in several serious neonatal diseases characterized by intense inflammation, such as hypoxic–ischemic encephalopathy (HIE), bronchopulmonary dysplasia, sepsis, and necrotizing enterocolitis.

Hypoxia–ischemia triggers NLRP3 activation in the central nervous system, particularly in microglia, and promotes neuroinflammation, with subsequent cell death and brain damage. Likewise, exposure to high levels of oxygen can cause brain injury in neonates via NLRP3 activation. Unconjugated bilirubin activates microglial NLRP3 inflammasomes, leading to neuronal damage and bilirubin-induced encephalopathy. Murine studies have demonstrated that neonatal activation of the NLRP3 inflammasome, triggered by inflammatory stimuli such as infection, can result in persistent neurodevelopmental alterations. These include a reduction in perineuronal net density and long-term behavioral changes observable in adulthood [17,18,19].

A devastating intestinal disease in premature infants, NEC, involves excessive inflammation mediated by NLRP3. Bacterial products like lipopolysaccharide from Cronobacter sakazakii activate the NLRP3 inflammasome, causing intestinal damage and cell death [20]. The pathophysiology of neonatal sepsis is characterized by biphasic immune dysregulation. It involves immune evasion mechanisms employed by encapsulated pathogens, cytokine overproduction, epithelial barrier disruption, and context-dependent inflammasome activation. Also, severe neonatal infections, such as those caused by *Escherichia coli*, can lead to the overactivation of the NLRP3 inflammasome, which can lead to a severe inflammatory response, leading to systemic organ damage [12,20,21,22].

In summary, all these studies emphasize the importance of the ontogenetic adjustment of neutrophil function and innate immune responses, which are beneficial for fetal growth in a biological environment. The reduction in neutrophil NLRP3 inflammasome activation is a major element in the inherent vulnerability to infections in neonates and preterm infants, leading to an increase in morbidity and mortality. Newborns, especially preterm infants, are at high risk of developing sepsis. These findings are closely linked to the immature development and functional responses of the neonatal immune system. Consequently, targeting the NLRP3 inflammasome could be a novel and noteworthy area for therapeutic advances in neonatal infection/sepsis and preterm birth.

### 3.3. NLRP3 Inflammasome Role in Intestinal Mucosal Immunity

NLRP3 plays an important function in anti-infection immunity and contributes to inflammatory conditions, including inflammatory bowel disease (IBD) and other immune-related intestinal disorders [23]. NLRP3 participates in maintaining the homeostasis and stability of the intestine and the immune reaction of the intestinal mucosa.

Also, during bowel inflammation, it manages the innate immune responses, contributing to the support of the ongoing inflammation and the disruption of the enteric barrier through an adaptation of the tight junction proteins and cell apoptosis [24].

The NLRP3 inflammasome resides in epithelial and immune cells. Its molecular structure regulates biological activity and detects microbial-derived ligands. The inflammatory response in the intestine is mediated by the activation of the NLRP3 inflammasome by bacterial toxins, ATP, reactive oxygen species, and other danger signals, initiating caspase-1 effector proteins to trigger proinflammatory elements such as IL-1 and IL-18. Moreover, caspase-1 triggers the gasdermin D protein, which is considered to be a crucial regulator of innate immunity [25]. It is assumed that proper activation of the NLRP3 inflammasome in intestinal epithelial cells supports intestinal stability and barrier integrity by inducing IL-18, which is essential for promoting epithelial cell production and the proliferation of intestinal endothelial cells [26].

### 3.4. Pathogenic Roles of the NLRP3 Inflammasome

Abnormal levels of the NLRP3 inflammasome and pro-inflammatory cytokines are linked to several gastrointestinal disorders, such as IBD [27]. The NLRP3 dysfunction is due to the loss of IL-10 signaling due to accumulated mTOR-induced mitochondrial damage [28]. The results reveal that NLRP3 inflammasome overactivation, triggered by disrupted epithelial barriers, can exacerbate inflammation and damage, leading to the overproduction of pro-inflammatory cytokines and pathogenic outcomes during disease progression.

### 3.5. Protective Roles of the NLRP3 Inflammasome

The NLRP3 inflammasome pathway is reportedly an indispensable part of promoting gut epithelial integrity and stability. Zaki et al. suggested that the NLRP3 inflammasome may exert a protective effect on intestinal homeostasis; furthermore, they observed that the lack of the downstream inflammasome aggravated pathological injury in gene-deficient mice [29]. Initiation of the NLRP3 inflammasome can accelerate a compensatory reaction for epithelial cell proliferation to preserve barrier integrity. Therefore, inflammasome deficiency causes an increase in the permeability of the intestinal barrier. The protective role of the NLRP3 inflammasome may be attributed to its capacity to modulate host defense in recognizing pathogens, signals, and clearing the infection. Also, it helps to keep intestinal homeostasis by controlling the integrity of the intestinal epithelium and regulating immune response to gut microbiota.

### 3.6. The Causes of Contradictory Results for the Role of NLRP3 Inflammasome

The opposing results of several studies on the role of the NLRP3 inflammasome may be attributed to alterations in laboratory and experimental circumstances, research types, or disease stages [30]. This contradictory role is seen in diseases such as IBD.

It is clear that the switch from a protective to a pathogenic role for the NLRP3 inflammasome, as in the context of IBD, is likely dependent on the nature and duration of the trigger, as well as the host’s genetic background. Understanding these roles is crucial for developing targeted therapies for IBD that can modulate inflammasome activity without compromising its beneficial functions. 

## 4. NLRP3 Inflammasome in Gastrointestinal Diseases Concerning the Pediatric Age Group

Several studies have shown the role of the NLRP3 inflammasome in the pathogenesis of digestive diseases. The stimulation of the NLRP3 inflammasome impacts gut homeostasis. Dysregulation of the NLRP3 inflammasome is a key factor in the pathogenesis of several gastrointestinal and accessory organ disorders, such as those in the stomach, intestine, liver, and pancreas. In stomach disease, it is involved with the persistent infection of Helicobacter pylori-related gastritis and gastric cancer. The NLRP3 inflammasome is also involved in inflammatory bowel disease, colorectal cancer, changes in the gut microbiome, and colitis and enteritis [31]. It is associated with liver diseases such as viral hepatitis, non-alcoholic fatty liver disease, alcoholic liver disease, cholestatic liver injury, drug-induced liver injury, autoimmune hepatitis, hepatic fibrosis, and hepatocellular carcinoma. The NLRP3 inflammasome participates in pancreatic disease, chronic pancreatitis, acute pancreatitis, severe acute pancreatitis, and pancreatic ductal adenocarcinoma.

### 4.1. Inflammatory Bowel Disease

Inflammatory bowel disease (IBD) is a chronic, recurrent intestinal disease. It results from an abnormal immune reaction to the intestinal microflora of the host and is marked by symptoms such as abdominal pain, bloating, and changes in bowel habits. Inflammatory bowel disease is influenced by multiple contributing factors such as environmental causes, infection-related mediators, and genetic vulnerability. IBD includes two major types: ulcerative colitis (UC) and Crohn’s disease (CD) [31], which can affect any segment of the gastrointestinal tract from the mouth to the anus. Evidence of genetic tendency among IBD patients is suggested, as well as malignancy.

Almost twenty percent of patients begin at less than 20 years old. In the pediatric age group, four percent of cases with IBD started less than 5 years ago, and 18% of cases started before age 10 years. The peak onset is during adolescence [32].

The differences in inflammasome response between pediatric and adult-onset IBD are not entirely recognized; however, evolving research suggests that there are key distinctions. Recent research has shown the inflammasome’s crucial role across all age groups [33]. There are some notable differences and distinctions between pediatrics, adolescents, and adults. These differences are often linked to the distinct disease presentation and underlying genetics of IBD at different ages. The key differences between pediatric-onset IBD, adolescent, and adult-onset IBD include predominantly the genetic and specific type of immune dysregulation, inflammasome response, and clinical behavior of the disease. Regarding the genetic and clinical presentation differences, in the pediatric age group, especially children under six years old, “called very early onset IBD (VEO-IBD)”, is often a more aggressive disease with a different phenotype compared to adult-onset IBD. A subset of pediatric IBD is thought to be a monogenic disease, caused by a single gene mutation, often related to primary immunodeficiencies. Defects in genes that regulate the inflammasome NLRP3 pathway can lead to uncontrolled inflammasome activation and severe early-onset inflammation. The early-onset IBD may be associated with a higher burden of common genetic variants and rarer, high-penetrance variants. The monogenic defects in IL-10 signaling mutations impair the anti-inflammatory responses, leading to uncontrolled inflammasome activation. Additionally, XIAP deficiency disrupts apoptosis and immune regulation, promoting excessive inflammation [33,34]. These are more common in children and can lead to dysregulated inflammasome activity; the NLRP3, caspase 1, and IL-1β may be active and promote severe mucosal inflammation and early-onset extraintestinal symptoms, such as arthritis and perianal disease [34,35,36]. Clinical features can present with unique symptoms like growth failure, delayed puberty, and a more extensive and severe disease than older-onset IBD [37]. Adolescents and adults usually have polygenic risk, which is influenced by environmental factors, such as diet or pollution, and microbiome dysbiosis and epithelial barrier dysfunction that lead to inflammasome activation [36]. Adolescent IBD often shares characteristics with both pediatric and adult IBD. The disease can be aggressive, and the unique challenges of this age group, such as delayed diagnosis and treatment adherence, can influence disease progression. The presentation of IBD in adults is typically more variable and is considered a multifactorial disorder resulting from a complex interplay of genetic, environmental, and immune factors. This means it is influenced by multiple genes, each with a small effect, rather than a single, dominant mutation. The genetic variants associated with adult IBD may affect inflammasome components, but they typically do not cause a severe, primary defect in the same way as monogenic forms of VEO-IBD [35].

To summarize data from the previous studies, polymorphisms in the NLRP3 gene and other inflammasome effectors have been linked to an increased susceptibility to IBD in general, but the impact and specific variants may vary in prevalence and severity between age groups. In some VEO-IBD cases, a defective NLRP3 or related pathway can be the direct cause of the disease, leading to a severe and treatment-resistant form. In adult IBD, dysregulation of the NLRP3 inflammasome is a prominent feature, but it is often a result of the interaction between genetic predisposition, environmental triggers, and the gut microbiota.

Pediatric IBD, especially VEO-IBD, offers a unique window into the direct genetic link to inflammasome dysfunction, providing valuable insights that can inform our understanding and treatment of IBD in all patients [14,34].

The clinical presentation in children often includes atypical symptoms like growth failure, delayed puberty, anemia, or perianal disease without classic GI symptoms. The disease tends to be more aggressive and extensive, with pancolitis being common in ulcerative colitis, while in adolescents and adults, the onset of IBD may show more typical IBD symptoms (e.g., abdominal pain, diarrhea) and have a disease course that begins to resemble adult-onset IBD [35,36,37]. The age-related differences in IBD, such as increased inflammation and growth issues, likely stem from differences in immune development and response. While not explicitly stated as an inflammasome difference, the immune system’s developmental stage in children could influence how inflammasomes are activated or how the body responds to inflammasome-driven inflammation [37,38].

A recent study by Granot et al. showed that pediatric-onset ulcerative colitis has a higher incidence of E3 extensive colitis and a higher need for systemic immunomodulators and biologics than adult-onset ulcerative colitis. Moreover, the pediatric-onset versus adult-onset Crohn’s disease revealed more L3 ileocolonic involvement and perianal disease phenotype, with higher exposure to immunomodulators and biologics. They reported significantly lower 15-year biologic-free survival among pediatric-onset IBD than with adult-onset IBD [39].

Over the past 20 years, growing evidence has suggested that Crohn’s disease and ulcerative colitis may be triggered by defective innate immune responses, leading to impaired clearance of antigens and/or pathogens and ultimately contributing to chronic inflammation and disease development. However, there remains a dispute about the protective roles of the NLRP3 inflammasome in IBD. Its association with IBD has been suggested, with studies reporting upregulation of NLRP3 and IL-1β in adult patients with ulcerative colitis (UC) [40] and Crohn’s disease [41]. Additionally, the activity and severity of the disease were perceived with increased excretion of the NLRP3 inflammasome and its effectors, such as IL-1β, in the mucosa of IBD patients. Elevated inflammasome gene expression and an abundance of immature intestinal macrophages have also been noted [42]. A molecular switch role of the NLRP3, endorsing an inflammatory phenotype in intestinal immune cells via IL-1β.

The progression of IBD is enhanced by the uncontrolled activation of the NLRP3 inflammasome and its key cytokines in experimental studies [26,43]. The early stimulation of NLRP3 in intestinal epithelial cells limits pathogen colonization and averts additional inflammation of the intestine [30]. Research indicates that a deficiency in the NLRP3 inflammasome increases susceptibility to experimental colitis in mice [29,44]. Furthermore, NLRP3 has a beneficial role in probiotic-centered treatment for colitis [45].

The conflicting findings regarding the role of NLRP3 in IBD may stem from variations in experimental protocols, available resources, differences in microbiota composition, and the genetic backgrounds of the mice used in the studies. The NLRP1 and NLRP3 inflammasome signaling pathways may regulate the immune mechanism of IBD in children by upregulating the expression of Caspase-1 and IL-1β. The study of Wang and Ma included 126 children with IBD: 32 children with Crohn’s disease and 94 children with ulcerative colitis. They found significantly higher mRNA expressions of NLRP1, NLRP3, Caspase-1, and IL-1β in CD or UC cases, which correlated with the severity of the disease. There was a significant positive correlation in the children with UC or CD between the mRNA expression levels of NLRP1, NLRP3, Caspase-1, and IL-1β with serum IgM and IgG levels. A positive correlation of the mRNA secretion of NLRP1 and NLRP3 inflammasomes with Caspase-1 and IL-1β was detected [46].

Genetic factors affect IBD by regulating the activation of inflammasomes; the CARD8 gene inhibits NLRP3 inflammasome assembly, interacts with NLRP3, and inhibits NLRP3 oligomerization caused by variant V44I that exacerbates colitis and Crohn’s disease [47].

Also, presented with VEO-IBD, the deficiency of the receptor-interacting protein kinase 1 (RIPK1) gene inhibits NLRP3 inflammasome activation upon lipopolysaccharide stimulation, participating in human colitis [48].

### 4.2. NLRP3 Inflammasome in Gut–Lung Interaction

Distinct connection concerning the pathology of IBD and pulmonary diseases. The use of advanced technology improved the accuracy of diagnosis of the link between gut and lung, such as microbiome sequencing and modern imaging like high-resolution computed tomography. Mansi et al. reported that up to 71% of children and adolescents with Crohn’s disease had abnormal bronchial hyperreactivity [49].

### 4.3. Celiac Disease

Celiac disease is an autoimmune disorder characterized by chronic inflammation that essentially affects the small intestine and is caused by eating gluten-containing foods. In a study done by Al-Assaf et al., they showed that gene expression of NLRP3 inflammasome in the peripheral blood of Iraqi children with Celiac disease was downregulated in the present samples, and it was accompanied by a decreased serum level of IL-1β [50].

## 5. NLRP3 Inflammasome and Gastrointestinal Tract Infection

It has been declared that the NLRP3 inflammasome is activated by several pathogenic organisms that invade the gastrointestinal tract, bacteria such as Helicobacter pylori, Campylobacter jejuni, Yersinia enterocolitica, and Clostridium difficile infections. Also, it is prompted by viral species such as adenovirus and enterovirus species in mice [51,52], as well as protozoal infections such as Entamoeba histolytica in children, by activating interleukins and Giardia [53,54].

### 5.1. Infectious Enteritis and Colitis

The NLRP3 inflammasome has an important role in infectious enteritis and colitis, and protection from intestinal pathogens. The NLRP3 inflammasome induces the secretion of pro-inflammatory cytokines and controls the majority of immune mechanisms necessary to resist infections, such as acidification of the phagosome in mice [55]. Augmentation of inflammation of the intestine is due to the activation of the NLRP3 inflammasome and IL-1β. There is evidence that induction of the NLRP3 inflammasome damages the intestinal barrier via the prevention of goblet cell maturation [56].

In animal models, the pathogens residing in the intestine can activate the NLRP3 inflammasome by several molecules, including hemolysin A generated by Proteus mirabilis. The stimulation of NLRP3 by *P. mirabilis* plays a crucial role in exacerbating colitis [57]. Clostridium difficile A and Clostridium difficile B toxins are produced by Clostridium difficile [58], and the Yersinia enterocolitica enterocolitis adhesin invasin postulates “signal I” for NLRP3 inflammasome activation, while the bacterial type three secretion system translocon constitutes “signal II” that initiates IL-18 maturation and secretion [59]. Clostridium difficile infections are linked to antibiotic-associated diarrhea and pseudomembranous colitis, with toxins that trigger the Pyrin inflammasomes and subsequent elevation of IL-1b-dependent tissue damage [60].

A study showed contradictory data regarding the protective role of NLRP3, as the mice deficient in NLRP3 or mice receiving a pharmacological inhibitor of NLRP3 had increased survival and amplified bacterial clearance compared with untreated mice due to decreased autophagy and increased phagocytosis by neutrophils [61]. The role of NLRP3 in Salmonella typhimurium infection is still intangible [62].

Ectopic colonization of Klebsiella aerogenes has been shown to activate the NLRP3 inflammasome in intestinal macrophages, leading to periodontitis in mice through robust IL-1β secretion [63]. The absence of NLRP3 or the inhibition of IL-1 eliminates the colitogenic effect of Klebsiella aerogenes in mice, highlighting the crucial role of the NLRP3–IL-1β axis in the development of oral commensal pathobiont-driven colitis [63].

Pathogen-induced inflammasome-mediated IL-1β secretion helps suppress pathogens by activating the host’s innate immune response, such as neutrophil recruitment through the production of adhesion molecules in endothelial cells. Some studies suggest that the NLRP3 inflammasome regulates the intestinal microbiota [29].

On the contrary, some studies report contradictory findings. They found that activation of the NLRP3 inflammasome in epithelial cells by G protein-coupled receptor signaling protects against dextran sodium sulfate-induced colitis [55]. Mice lacking NLRP3 are more prone to dextran sodium sulfate-induced colitis [29,30,44]. These studies observed that NLRP3 activation leads to IL-18 secretion, which guards against colitis. IL-18 signaling helps maintain homeostasis in the commensal microbiota by inhibiting the growth of pathobionts [30]. This contradiction could be due to the variances in the gut microbiota among the different studies. The stimulation of the NLRP3 inflammasome by precise pathobionts could play a critical role in either triggering or exacerbating intestinal inflammation.

### 5.2. Helicobacter Pylori

Helicobacter pylori (HP) is a damaging bacterium that is located in the gastric mucosa and is frequently acquired in childhood with some grades of gastric mucosal inflammation, which may progress from chronic gastritis to adenocarcinoma [64]. The WHO classifies *HP* as a Group 1 carcinogen [51]. The NLRP3 inflammasome and HP infection augment each other in an endless circle. The amount of NLRP3 and gasdermin D protein was considerably elevated in the stomach of HP patients. It was proven that during the initial phases of HP infection, the NLRP3 inflammasome is activated through the release of hsamiR-223-3p and IL-10 in cultured and primary human immune cells [65]. HP also imparts a secondary signal mandatory for NLRP3 inflammasome activation in isolates in human immune cells, including K+ efflux and reactive oxygen species release, which control the increase in IL-1β secretion. Elevated levels of mature IL-1β during *HP* infection can contribute to the development of atrophic gastritis and cancer [66].

### 5.3. Protozoan Infections

NLRP3 has also been involved in protozoan infections, as it was found critical in identifying the Entamoeba histolytica invasion and in escalating a vigorous inflammatory reaction. *Giardia duodenalis* is a frequent cause of diarrhea among children in developing countries. Manko-Prykhoda et al. stated that a new NLRP3-modulatory mechanism regulates the severity of enteric diseases during co-infections with *Giardia* spp. and A/E enteropathogens; they reported a decrease in colitis, bloody soft stool, bacterial invasion, and weight loss in co-infected mice [53].

### 5.4. Viral

The involvement of NLRP3 in the COVID-19 gastrointestinal affection was shown by a study by Sinaei et al. in infants and children aged 1 month to 16 years; the presentations were a variety of gastrointestinal symptoms, such as vomiting, diarrhea, and abdominal pain [67]. The NLRP3 inflammasome may be stimulated by several elements of SARS-CoV-2. The S protein is associated with angiotensin-converting enzyme 2, which leads to NLRP3 inflammasome activation [68]. Remarkably, the SARS-CoV-2 viral proteins viroporin 3a and ORF8b were suggested to activate NLRP3, and genetic modification of these viral proteins was related to variation in the severity of clinical pictures [69].

Multisystem Inflammatory Syndrome in Children (MIS-C) has gastrointestinal symptoms, higher levels of markers of myocardial injury, elevated ferritin, and frequently manifests with shock. Furthermore, there is evidence of inflammasome over-activation in MIS-C with marked elevation of IL-6 and IL-18 [70].

## 6. Inflammasome and Neonatal Gastrointestinal Disorders

Necrotizing enterocolitis

One of the emergencies in the neonates admitted to the NICU is necrotizing enterocolitis (NEC) [71]. The NLRP3 inflammasome plays a significant role in the pathogenesis of NEC. It has been assumed that the restraint of stimulation of NLRP3 could have protective consequences on NEC0. The inflammasome acts as a microbial sensor, and its activation by the commensal microbiota may be a factor in the pathophysiological outcomes of NEC. NLRP3 inflammasome activation was reported to be an important regulator in the development of NEC. Yin et al. found that NLRP3 inflammasome and downstream inflammatory factors, such as IL-1β and IL-18, were increased in NEC human and mouse intestinal tissues [72]. There was an elevation of NLRP3, pro-Caspase-1, and Caspase-1 p10 in the mice with NEC. In neonates, NLRP3 and Caspase-1 were increased in NEC, and they were expressed both in the epithelium and lamina propria. These data demonstrated that NLRP3 inflammasome enzymatic protein caspase-1, as well as its downstream IL-1b and IL-18, were increased in NEC [72].

The intensified expression of NLRP3 in the hippocampus and cerebral cortex proved the role of the NLRP3 inflammasome in the pathogenesis of NEC-associated brain injury. Also, high levels of caspase-1 and mature IL-1β were demonstrated in the brain of the NEC animal model [73]. IL-1β facilitates pro-inflammatory cytokine levels, stimulates microglia, and interrupts the blood–brain barrier, consequently participating in a diversity of neuroinflammatory diseases [74].

Zhu et al.’s study discloses a possible relation between the activation of NLRP3 inflammasome, intestinal and brain injury, and long-term intellectual dysfunction. They found a significant rise in the IL-1β levels of the intestine of the experimental NEC in mice, which is related to increased activity of the inflammasome NLRP3. IL-1β triggers damage to the intestinal mucosal barrier, leading to neutrophil recruitment to the damaged site and endorsing macrophage stimulation. They studied the effect of blockage of NLRP3 inflammasome activation to detect if it can ameliorate acute inflammatory injury, brain injury, and long-term cognitive impairment induced by necrotizing enterocolitis in mice. They showed that MCC950, which is a selective small molecule inhibitor of NLRP3, markedly reduced the overall mortality of the NEC mouse model, downregulated proinflammatory cytokine expression (mature IL-1β, IL-6, and TNF-α), and markedly improved the severity of histological damage in the intestines of NEC mice. MCC950 has a protective effect from NEC and NEC-induced acute brain damage, as well as their long-term cognitive impairment. It is a promising new therapeutic modality for infants suffering from NEC [73].

Macrophage α7nAChR activation mitigates NLRP3 inflammasome activation by modulating mTOR phosphorylation and subsequently alleviates intestinal inflammation and injury in NEC. Shen et al. demonstrated that the expression of α7nAChR was elevated in both the mucosal and submucosal layers of the intestines in neonatal NEC patients and mouse models. In vitro experiments showed that treatment with the α7nAChR agonist PNU-282987 significantly suppressed the upregulation of NLRP3, activated caspase-1, and IL-1β in macrophages. Additionally, administration of the mTOR inhibitor rapamycin also inhibited the expression of these inflammatory markers. Furthermore, PNU-282987 treatment led to a reduction in both the proportion of intestinal macrophages and the extent of intestinal injury [75].

Additionally, the microRNA miR-146a-5p, another regulator of the NLRP3 inflammasome, was found to suppress downstream inflammatory mediators and the membrane expression of CLIC4 in NEC patients and experimental models. miR-146a-5p contributed to the reduction of inflammation and intestinal damage in NEC-affected tissues. Its expression was notably elevated and predominantly localized within macrophages in the affected intestinal regions. In vitro, the miR-146a-5p mimic was the only agent that inhibited the downstream inflammatory factors of the NLRP3 inflammasome, as well as the expression of its upstream protein, chloride intracellular channel protein 4 (CLIC4), in the cellular membrane of the THP-1 cell line. MiR-146a-5p overexpression via adenovirus transfection reduced CLIC4 cellular membrane expression and inhibited NLRP3 downstream factors, resulting in an increase in vivo. Following transfection with the miR-146a-5p adenoviral vector, NEC mice exhibited improved survival rates and reduced intestinal damage [76,77].

Macrophages in which NLRP3 is activated infiltrate the NEC intestine and serve as key mediators of inflammation and drive pyroptotic cell death, amplifying inflammatory cascades. It correlates with intestinal damage severity and mortality in NEC models.

## 7. Inflammasome and Hepatic Disorders

Inflammasomes are extracted from the parenchymal and non-parenchymal cells of the liver in response to harmful activators. Inflammasomes are dynamic in hepatocytes, liver sinusoidal endothelial cells, hepatic stellate cells, and macrophages and are involved in the pathogenesis of acute liver injury, chronic liver diseases with augmented gut permeability, infectious diseases of the liver, including hepatitis C virus infection, and schistosomiasis. It is also associated with drug-induced liver injury, such as acetaminophen, ischemia/reperfusion, or endotoxin.

### Viral Hepatitis

Viral hepatitis is one of the most common infections associated with hepatic disorders in the pediatric age group. Hepatitis B virus (HBV) infection in infants and children is often associated with few or no symptoms but poses a high risk of developing into a chronic condition [78]. The hepatic concentrations of NLRP3, apoptosis-associated speck-like CARD-domain containing protein, and IL-1b from chronic hepatitis B patients were associated with HBV-DNA intensity [31]. NLRP3, IL-18, IL-1β, and caspase-1 were significantly increased in hepatic tissues of patients with HBV-associated acute-on-chronic liver dysfunction. Likewise, hepatitis B core antigen was found in experimental studies to induce the expression of NLRP3 inflammasome and IL-1β [79]. Therefore, persistent infection with HBV triggers the NLRP3 signaling pathway, resulting in damage to hepatic tissues by the expression of cytokines such as IL-1β and IL-18. Adult patients who suffered from chronic hepatitis showed high values of IL-1β. Following Hepatitis C infection, apoptosis-associated speck-like protein containing a caspase (ASC) interacts with NLRP3 and dissociates from Golgi-resident protein immunity-related GTPase M, leading to Golgi breakup. This mechanism enhances the multiplication of HCV, which promotes hepatic chronic inflammation [80].

Also, the elements of the hepatitis E virus strongly activate the NLRP3 inflammasome in primary macrophages and macrophage cell lines. Remarkably, inflammasome activation counteracts interferon reaction to enable HEV reproduction in macrophages of patients and rabbits [81,82]. Future approaches to restrain the NLRP3 inflammasome or its inflammatory cytokines could offer treatment to alleviate liver inflammation alongside antiviral treatments.

## 8. Pharmacological Inhibitors for NLRP3 Inflammasome

Pharmacological inhibitors that target the NLRP3 inflammasome may be a better alternative for the treatment of NLRP3-associated diseases [3,83]. Inhibitors often target the “upstream signaling” that activates the NLRP3 inflammasome, rather than targeting the “NLRP3 protein itself”. This indirect approach can limit their effectiveness and may cause unforeseen side effects. As a result, many scientists believe gene editing is a more effective strategy for modulating NLRP3 activity and are conducting extensive research on this method. The NLRP3 inflammasome can be inhibited through direct and indirect inhibitors [84]. Inhibition of NLRP3-driven inflammation can be achieved directly or indirectly through targeting signaling pathways, such as transcription and oligomerization, inhibition, or Gasdermin D cleavage inhibition. Indirect inhibitors for the NLRP3 inflammasome reduce the activation of the NLRP3 inflammasome in three ways: by inhibiting the protein expression of the NLRP3 inflammasome, blocking the upstream signaling pathway of the NLRP3 inflammasome, and inhibiting the post-translational modification of regulatory proteins of NLRP3. The indirect inhibitors include glyburide, 16673-34-0, JC124, and FC11A-2. Also, there are inhibitors for the constituents of the NLRP3 inflammasome that include parthenolide, VX-740, VX-765, Bay 11-7082, and β-hydroxybutyrate (BHB).

Direct inhibitors of the NLRP3 inflammasome have the benefits of abolishing processes and inhibiting NLRP3 inflammasome activation. The direct inhibitors include diaryl sulfonylurea-containing compound MCC950, 3,4-Methylenedioxy-b-nitrostyrene, CY-09, OLT1177, tranilast, and oridonin [3,83]. MCC950 has shown promise in reducing inflammation and tissue damage in animal models of neonatal brain injury and NEC.

Additionally, provitamin A can blend with the pyrin domain (PYD) part of NLRP3, inhibit the activation of the NLRP3 inflammasome, and directly reduce the expression level of IL-1β. CY-09 also inhibits the ATPase activity of NLRP3. Moreover, among the direct inhibitors, 3,4-methylenedioxy-β-nitrostyrene directly binds to the leucine-rich repeat domain (LRR) and nucleotide-binding and oligomerization domain (NACHT) of NLRP3 and subsequently restrains the activation of the NLRP3 inflammasome by inhibiting the ATPase activity of NLRP3. Bay11-7082 inhibits the activation of the NLRP3 inflammasome by alkylating the ATPase active region of NLRP3 and inhibiting the NF-ĸB signal. Among these NLRP3 inhibitors, tranilast, β-hydroxybutyrate, paeoniflorin, coptisine, BAY 11-7082, and Bifidobacterium longum have a possible role in IBS symptom management [84].

Third-generation genome editing tool targeting the NLRP3 inflammasome, specifically clustered regularly interspaced short palindromic repeats (CRISPR)/CRISPR-associated protein 9 (Cas 9), could precisely destroy or repair pathogenic genes through a single guide (g)RNA-directed Cas 9 nuclease [83].

The preclinical studies in neonatal models exploring the role of aluminum-based adjuvants (alum) have revealed that the prophylactic activation of the NLRP3 inflammasome augments innate immune responses and increases survival in neonatal sepsis models. This action is independent of B and T lymphocytes, proposing a mechanism of trained immunity that improves pathogen resistance in neonates [85,86]. Among these mechanisms, the NLRP3 inflammasome stands out as a promising therapeutic target. Although still in the preclinical stage, alum acts as a possible immunomodulatory therapy to restore immune equilibrium and improve outcomes in neonates [85].

There are still no available therapeutic drugs in clinical use that specifically target the NLRP3 inflammasome. A few inhibitors have been promoted to clinical trials of inflammatory diseases in adults. Still, there are challenges in relation to specificity, methods of delivery, unproven efficacy and safety, and the need for more verification [83], especially in the pediatric age group.

Future research must benefit from new data regarding the structure of NLRP3 and develop direct inhibitors with better specificity and effectiveness. The nanobodies are recently being studied as therapeutic drugs; they have high specificity, stability, and low toxicity [87].

In summary, all of the previous promising inhibitors have not been approved by the Food and Drug Administration or other agencies. However, IL-1β inhibitors could be utilized in clinical trials. More research is needed to develop suitable inhibitors with better pharmacokinetic properties that can reach the specific organs with no side effects and be more cost-effective.

Food bioactive compounds, such as NLRP3 inflammasome modulators, encompass several chemical classes, including polyphenols, organosulfurs, terpenes, fatty acids, proteins, amino acids, saponins, sterols, polysaccharides, carotenoids, vitamins, and probiotics. These compounds have been shown to possess NLRP3 inflammasome-modulating activity through in vitro and in vivo assays. It mainly demonstrates an anti-NLRP3 inflammasome activity. Plant foods are particularly rich in important bioactive compounds. Every compound has different effects on the inflammasome pathway and inflammatory response. This confirms the significance of the nutritional/food model as a whole rather than any single nutrient or functional compound [88].

Moreover, curcumin can inhibit NLRP3 inflammasome activation and reduce the production of pro-inflammatory cytokines, such as IL-1β and IL-18. Curcumin is a promising natural anti-inflammatory agent for treating sepsis [89]. Table 1 shows the therapeutic implications.

## 9. Quality, Strengths, and Limitations of the Studies

There is a vast amount of high-quality experimental and animal research related to the role of the inflammasome. These studies showed the unique role of NLRP3 in gastroenterology disorders; however, when it comes to the pediatric population, a few studies have been conducted, especially in relation to hepatic and infectious diseases.

Several limitations exist; although a considerable number of good-quality studies have been conducted on childhood IBD, no systematic analysis of the current studies has been conducted. A few investigations showed that the link between the genetic susceptibility of NLRP3 inflammasome-associated genes and the molecular regulation of the NLRP3 inflammasome is of particular importance. The studies did not precisely verify the NLRP3 inflammasome’s differential role as either a pathogenic or a protective function during the course of the disease. There are also limitations for the biomarkers that can identify the protective or injurious role of NLRP3.

Moreover, it is currently clear that targeting activation of the NLRP3 inflammasome and the related endpoints (IL-1β, IL-18, pyroptosis) will provide new insights into the development of novel therapeutic options in IBD and other diseases mediated by NLRP3; however, there are still no evidence-based studies in relation to the pediatric age group.

These limitations underscore the urgent need for longitudinal, patient-centered studies incorporating immunophenotyping and real-time biomarker assessments, especially in the pediatric age group, to evaluate the efficacy and safety of inflammasome-based therapies. Without such data, there is a risk of misapplication, leading to ineffective or even harmful outcomes. The evidence gaps are shown in Table 2. 

## 10. The Future Direction

The NLRP3 inflammasome is involved in multiple gastrointestinal and hepatic diseases among the pediatric age group. However, exclusive research is in the initial phase. Future studies to elucidate the NLRP3 inflammasome’s protective and or pathogenic role in intestinal disorders are needed.

Future studies may reveal what is better: direct inhibition or indirect inhibition of the NLRP3 inflammasome. More extensive research is needed for the role of the anti-inflammatory effect of BHB in inhibiting the NLRP3 inflammasome and subsequent management of the NLRP3-mediated inflammatory diseases. The relation to gut probiotics is also still not well clarified.

The NLRP3/caspase-1/IL-1β/IL-18 axis is a good target for treating inflammasome-related diseases; a potential study of these axis inhibitors, bioavailability, effectiveness, and safety may show the possibility of their therapeutic impact [84].

Precise information is required regarding the mode of action of the inflammasome inhibitors, such as the synthetic small molecules, phytochemicals, organic compounds, and probiotics, e.g., as a therapeutic alternative for IBS management [90].

Moreover, prospective research related to BHB to confirm its effect in inhibiting NLRP3 inflammasome activation and regulating intestinal pro-inflammatory Th17 cells could be beneficial [65]. Pursuing NLRP3 using gene editing techniques, such as exosomes and miRNA, may present some concepts for future direction [83].

## 11. Conclusions

This article consolidates previously dispersed research into a cohesive and comprehensive review. The structure of NLPR3 and significant current known mechanisms in innate and adaptive immunity show an important contribution to multiple gastrointestinal and hepatic disorders in neonatal and pediatric age groups. The NLRP3 inflammasome is stimulated by a variety of stimuli. The activation of the NLRP3 protein engages the ASC protein, which recruits the procaspase-1, leading to initiation, maturation, and secretion of inflammatory cytokines and pyroptosis.

The NLRP3 inflammasome has a dual role as a protective or injurious cause for various gastrointestinal disorders, such as IBD and NEC. The overstimulation or dysregulated activity leads to pathological injury to the intestinal mucosa. There are multiple gaps in our understanding of various aberrant cellular and molecular events that participate in the activities and response of NLRP3. There are no precise biomarkers that identify the protective and pathological state of the disease.

Current studies have revealed various therapeutic inhibitors of the NLRP3 inflammasome pathways using animal models, either through direct inhibition of the NLRP3 protein or other components and products of the inflammasome. Table 3 shows the current pediatric-related studies. 

## Figures and Tables

**Table 1:** The protective vs. pathogenic roles of the NLRP3 inflammasome in neonatal and pediatric gastrointestinal and liver diseases.

Disease	Protective Role of NLRP3	Pathogenic Role of NLRP3	Therapeutic Implication
Neonatal Necrotizing Enterocolitis	It serves to eradicate infectious agents while triggering a repair response through the action of IL-1β and IL-18.	Uncontrolled and excessive activation leads to epithelial injury and the development of systemic inflammation.	Treatment strategies could include using IL-1 receptor antagonists (like anakinra) and administering probiotics to adjust the gut microbiota. Neonatal-specific dosing and safety data are currently unavailable.
Pediatric Inflammatory Bowel Disease and Crohn’s Disease	It plays a role in both mucosal protection and microbial homeostasis.	Overactivation causes chronic inflammation and barrier dysfunction.	MCC950, which is an NLRP3 inhibitor. Dietary interventions.
Viral Hepatitis and Autoimmune Hepatitis	It may facilitate the elimination of infected or compromised hepatocytes.	Sustained activation leads to immune dysregulation and liver injury.	NLRP3 inhibitors and IL-1 β blockade.
Gut–Liver Axis Disorders	It maintains the gut barrier and prevents microbial translocation.	Interruption of the gut barrier predisposes to generalized inflammation and liver damage.	Probiotics; bile acid modulators; NLRP3 inhibitors.
Giardia duodenalis	It boosts the immune response driven by IL-1β and simultaneously decreases the number of parasites as well as the damage to the intestines.	Excessive activation may contribute to inflammation and epithelial damage.	Targeting extracellular vesicles or modulating NLRP3 may reduce pathogenicity; probiotics and IL-1 blockers could be explored.
Entamoeba histolytica	Acts as a sensor for invasive contact; it initiates rapid host defense.	Drives excessive inflammation and tissue destruction in amebic colitis.	Blocking integrin-NLRP3 signaling may reduce tissue damage; anti-inflammatory agents could be beneficial.
Helicobacter pylori	Controlled activation may help contain infection and prevent excessive inflammation.	Suppression of NLRP3 allows immune evasion and promotes gastric pathology.	Inhibiting mitophagy or regulating NLRP3 activity may enhance immune clearance; IL-1β modulation is a potential strategy.

**Table 2:** Evidence gaps in NLRP3 inflammasome research in pediatric GI and liver diseases.

Item	Identified Evidence	Gaps in the NLRP3 Role in Pediatrics
Diseases	NLRP3s contribute to mucosal injury in IBD. NLRP3 activation is linked to IL-1β/IL-18 release and immune dysregulation. NLRP3’s role is recognized in MIS–C and NEC. Perinatal complications; preclinical studies, in mouse models, have shown the NLRP3 inflammasome is linked to preterm labor/birth and adverse neonatal outcomes. Evidence from children, adults, and animal models.	The information regarding the activation of NLRP3 in neonatal and pediatric-specific conditions, including biliary atresia, neonatal cholestasis, and pediatric autoimmune hepatitis, is limited. The mechanism related to NLRP3 in neonatal cholestasis is not known. The modulation role of neonatal NEC is not clear. The exact triggers for IBD, regulatory procedures, and progression patterns are unclear. Lack of studies related to the mechanism of injury of pediatric non-alcoholic fatty liver disease. Underexplored role of autoimmune hepatitis in pediatrics. Pediatric models for liver diseases are deficient. No adequate, precise biomarkers for liver diseases. Lack of clinical studies for the role of NLPR3 in perinatal complications. Genetic susceptibility of NLRP3 inflammasome-associated genes and the molecular regulation of the NLRP3 inflammasome.
Immune System Response	NLRP3 activation mechanisms are characterized in animals and adults.	An immature immune system is primed to incomplete recognition of how neonates’ and children’s immune systems adjust and react to NLRP3 activation.
Activation Mechanisms	Both canonical and non-canonical pathways are outlined, with potassium efflux, mitochondrial dysfunction, and lysosomal damage identified as primary activating factors. NLRP3 contributes to hepatocyte death by inducing pyroptosis and promoting the release of inflammatory cytokines.	-Pediatric-specific triggers and regulatory checkpoints remain inadequately characterized, highlighting the need for further research into age-dependent mechanisms of inflammasome activation.
Inflammasome Interaction with Intestinal Microbiota	Dysbiosis activates the NLRP3 inflammasome, contributing to both gastrointestinal and hepatic inflammation.	-Intestinal microbiota is different in neonates and children and can affect the response to the inflammasome and vulnerability to diseases. -Scarcity of longitudinal pediatric studies exploring microbiota development and its influence on NLRP3 activation. -Underscoring the need for age-targeted research, especially for inflammasome–microbiota interactions in IBD.
Protective vs. Pathogenic Role	NLRP3 can be both protective and abolish pathogens and suppress infection. It is also pathogenic and increases the inflammatory response.	-Few pediatric studies have investigated the dual roles of NLRP3, whether protective or pathological, in gastrointestinal and liver diseases. -Currently, no reliable biomarkers exist to distinguish the protective from injurious role of NLRP3 or to guide precise therapeutic strategies.
Diagnosis/monitoring Biomarkers	IL-1β, IL-18, and gasdermin D are known markers of NLRP3 activation in adults and animal studies.	There are no age-specific biomarkers available for early diagnosis or monitoring of NLRP3-related responses. The proven pediatric-specific studies on biomarker responses to NLRP3 remain scarce.
Therapeutic Targeting	Several direct and indirect inhibitors of NLRP3 and IL-1 blockers (e.g., anakinra) show promising results; MCC950 and other small molecules are under investigation in animal and early adult studies.	Pediatric susceptibility and inflammasome response to drugs are poorly defined due to inadequate drug safety, dosing, and efficacy. Data are deficient; no approved NLRP3-targeted drugs for children yet. A few preclinical studies in pediatrics have been conducted in relation to targeting activation of the NLRP3 inflammasome and the related endpoints (IL-1β, IL-18, pyroptosis). Pediatric trials focusing on pharmacogenomic and pharmacokinetic aspects of targeted therapies in children and neonates are lagging, limiting progress toward personalized treatment approaches. Use of food bioactive compounds.
Experimental Models	Mouse models have clarified NLRP3’s role in adult GI/liver diseases.	Few neonatal or pediatric animal models (mice, rabbits) exist to study NLRP3 behavior 0000in early gut/liver life diseases. Lack of age-appropriate pediatric models to study the inhibitory drugs.
Inflammasome Crosstalk	NLRP3 interacts with other inflammasomes (e.g., NLRP1, AIM2).	Crosstalk in pediatric GI/liver diseases is poorly understood. The relation to the brain axis and the respiratory system needs more investigation.

**Table 3:** The pediatric-related studies.

N	Author	Topic	Study Design	Published Year
1.	Zahid, et al. [3]	Pharmacological Inhibitors of the NLRP3 Inflammasome	Review—all age groups	2019
2.	Song, et al. [6]	Biological functions of NLRP3 inflammasome: A therapeutic target in inflammatory bowel disease.	Review—all age groups	2021
3.	Hoffman H.M. [11]	Inflammasome and IL-1beta-mediated disorders.	Review—all age groups	2010
4.	Mohamed, et al. [12]	The role of nod-like receptor family pyrin domain-containing 3 (NLRP3) inflammasome in the diagnosis of late-onset neonatal sepsis	Original article	2022
5.	Hanaei, et al. [13]	Association of NLRP3 single nucleotide polymorphisms with ulcerative colitis: A case-control study	Original article—all age groups	2018
6.	Sharma, et al. [15]	Impaired NLRP3 inflammasome activity during fetal development regulates IL-1β production in human monocytes.	Original article	2015
7.	Kenichiro Motomura, et al. [16]	Fetal and maternal NLRP3 signaling is required for preterm labor and birth.	Original article	2022
8.	Wackerbarth, et al. [17]	Neonatal neutrophils exhibit reduced NLRP3 inflammasome activation	Original article	2025
9.	Kiser, et al. [18]	NLRP3 inflammasome: a key player in neonatal brain injury	Original article	2025
10.	Shi, et al. [20]	NLRP3 activation in macrophages promotes acute intestinal injury in neonatal necrotizing enterocolitis	Original article	2024
11.	Zaki, et al. [30]	The Nlrp3 inflammasome: contributions to intestinal homeostasis.	Review—all ages	2011
12.	Qiang, et al. [31]	NLRP3 inflammasome in digestive diseases: From mechanism to therapy	Review—all age groups	2022
13.	Abramson, et al. [32]	Incidence, prevalence, and time trends of pediatric inflammatory bowel disease in Northern California, 1996 to 2006	Original article	2010
14.	Guner, et al. [33]	Genetic Variants in Early-Onset Inflammatory Bowel Disease: Monogenic Causes and Clinical Implications.	Original article	2025
15.	Nambu, et al. [34]	A Systematic Review of Monogenic Inflammatory Bowel Disease	Systematic review	2022
16.	Vuijk, et al. [35]	Considerations in Paediatric and Adolescent Inflammatory Bowel Disease	Review	2024
17.	Rosen et al. [36]	Inflammatory Bowel Disease in Children and Adolescents	Review	2015
18.	Ruel, et al. [37]	IBD across the age spectrum: is it the same disease?	Review—all age groups	2014
19.	Alwassief, et al. [38]	Transitioning Pediatric Patients with Inflammatory Bowel Disease: Key Considerations for Adult Gastroenterologists.	Review	2025
20.	Granot, et al. [39]	Differences in disease characteristics and treatment exposures between paediatric and adult-onset inflammatory bowel disease using a registry-based cohort.	Original article	2024
21.	Wang, et al. [46]	Role of NLRP1 and NLRP3 inflammasome signaling pathways in the immune mechanism of inflammatory bowel disease in children.	Original article	2020
22.	Mao, et al. [47]	Loss-of-function CARD8 mutation causes NLRP3 inflammasome activation and Crohn’s disease.	Original article—all age groups	2018
23.	Li, et al. [48]	Human RIPK1 deficiency causes combined immunodeficiency and inflammatory bowel disease.	Original article	2019
24.	Mansi, et al. [49]	Bronchial hyperresponsiveness in children and adolescents with Crohn’s disease.	Original article	2000
25.	Al-Assaf, et al. [50]	Gene Expression of NLRP3 Inflammasome in Celiac Disease of Iraqi Children.	Original article	2021
26.	Abdul-Aziz [53]	Molecular study and determining the levels of some interleukins in children with Entamoeba histolytica.	Original article	2025
27.	Nguyen et al. [64]	Helicobacter pylori Infections in Children.	Review	2023
28.	Sinaei et al. [67]	Gastrointestinal and hepatic manifestations among hospitalized COVID-19 children.	Original article	2025
29.	Nakra, et al. [70]	Multi-System Inflammatory Syndrome in Children (MIS-C) Following SARS-CoV-2 Infection: Review of Clinical Presentation, Hypothetical Pathogenesis, and Proposed Management	Review	2020
30.	ELMeneza, et al. [71]	Inter-Alpha Inhibitor Proteins as a Predictor of Necrotizing Enterocolitis in Newborn Infants.	Original article	2023
31.	Shen, et al. [75]	Macrophage α7nAChR alleviates the inflammation of neonatal necrotizing enterocolitis through mTOR/NLRP3/IL-1β pathway.	Original article	2024
32.	Lin, et al. [78]	Hepatitis B: Immunization and Impact on Natural History and Cancer Incidence	Review	2020
33.	Chen, et al. [76]	The role of NLRP3 inflammasome in necrotizing enterocolitis.	Original article	2025
34.	Chen, et al. [77]	MiR-146a-5p Mimic Inhibits NLRP3 Inflammasome Downstream Inflammatory Factors and CLIC4 in Neonatal Necrotizing Enterocolitis.	Original article	2021
35.	Speer, et al. [87]	Neonatal Murine Escherichia Coli Sepsis Model Demonstrates That Adjunctive Pentoxifylline Enhances the Ratio of Anti- vs. Pro-Inflammatory Cytokines in Blood and Organ Tissues	Original article	2020
36.	Rincon et al. [88]	Aluminum Adjuvant Improves Survival via NLRP3 Inflammasome and Myeloid Non-Granulocytic Cells in a Murine Model of Neonatal Sepsis.	Original article	2021
